# Extracorporeal Acoustic Shock Waves to Treat Complications of Polymethylmethacrylate

**DOI:** 10.1007/s00266-024-04586-x

**Published:** 2024-12-16

**Authors:** Mario Goisis, Sara Zecchetto, Sheila Veronese, Lindsey Alejandra Quintero Sierra, Riccardo Ossanna, Paolo Bernardi, Maria Maddalena Nicoletti, Sima Khabouri, Andrea Sbarbati

**Affiliations:** 1 De Clinic, Viale Regina Giovanna 39, 20129 Milan, Italy; 2 Aesthetic Medicine Clinic - Dr. Sara Zecchetto, Piazza del Popolo 16, 37132 Verona, Italy; 3 Section of Anatomy and Histology, Department of Neuroscience, Biomedicine, and Movement, University of Verona, P.le L.A. Scuro 10, 37134 Verona, Italy; 4 Department of Precision Medicine, University of Campania “Luigi Vanvitelli”, Piazza Luigi Miraglia 2, 80138 Naples, Italy

**Keywords:** Shock waves, AWT, Polymethylmethacrylate, Filler, PMMA, Dermagraft

## Abstract

**Background:**

Polymethylmethacrylate (PMMA) fillers are permanent fillers known for their possible side effects. In case of complications, the only possible treatment is surgical removal, followed by procedures to minimize resulting deformity. The aims of this study were (1) to analyse the morphology of the PMMA material in the nodules, (2) to demonstrate that treatment by acoustic wave therapy (AWT) can help the removal of the nodules, and (3) to present an easy procedure to solve deformities.

**Methods:**

The data of 9 patients who underwent AWT, surgical PMMA removal, and deformity correction by enriched nanograft and dermagraft injections between April 2021 and May 2024 were presented. The leftover aspirated material was analysed by histology and scanning electron microscopy.

**Results:**

AWT resulted in no complications in all patients. After removal and correction surgeries, minor complications were observed in 5 cases. All the patients were delighted with the aesthetic outcome. In all patients, an important improvement of the deformities, with correction of the volume of the face, was observed. A substantial resolution of the initial clinical symptoms was documented. The ultrastructural analysis highlighted that PMMA appears in the form of laminar or prismatic formations with a paracrystalline structure.

**Conclusions:**

AWT acted directly on PMMA to facilitate its removal and reduce fibrosis around the PMMA filler. The lack of volume correlated with PMMA removal was resolved with the enriched nanograft and dermagraft injections, which led to very satisfying aesthetic results.

**Level of Evidence V:**

This journal requires that authors assign a level of evidence to each article. For a full description of these Evidence-Based Medicine ratings, please refer to the Table of Contents or the online Instructions to Authors www.springer.com/00266.

## Introduction

Aesthetic improvement has been a human desire throughout recorded history and in all cultures [1].

Since a few years ago, surgical techniques have dominated the facial rejuvenation field. Currently, with the availability of nonsurgical procedures that offer cosmetic improvements and the convenience of minimal downtime, the trend is towards less invasive procedures and prophylactic interventions. The face is one of the main protagonists of aesthetic treatments. For instance, restoring facial volume using fillers can rebalance facial proportions, increase symmetry, reduce wrinkles and volume loss, and produce a younger, healthier appearance [2].

Although soft tissue augmentation dates back to over a century ago, when autologous fat began to be used, injectable fillers entered mainstream aesthetic medicine when bovine collagen injections were developed in the 1980s. Subsequently, minimally invasive injections of cosmetic fillers quickly spread to the field of aesthetic medicine [3].

Fillers can be classified in various ways. Based on the source, they may be classified as autologous, biological, or synthetic. Depending on the duration of the cosmetic benefit, these can be short-lived (less than 3 months), medium-lasting (3–12 months), long-lasting (12–24 months), or very long-lasting (more than 24 months). Using reversibility as a classification criterion, fillers can be classified as very rapidly reversible, slowly biodegradable but not reversible, or non-biodegradable [4].

Hyaluronic acid and calcium hydroxyapatite fillers are temporary fillers wholly degraded by the surrounding tissue after a few months. In contrast, permanent fillers, such as those made from silicon oil and polymethylmethacrylate (PMMA) microspheres, are not biodegradable. Since these latter fillers remain in place and are not degraded, the side effects related to their presence can be severe and irreversible [3]. Many authors have described the complications of these fillers. Most complications are produced by a low-grade bacterial infection and foreign body reaction and lead to fibrosis [5]. According to the causes of the complications, their management has mainly focused on the use of anti-inflammatory drugs and antibiotic treatment.

Obviously, temporary fillers usually cause temporary side effects, while side effects related to permanent fillers can last forever [6]. Consequently, in case of complications of permanent fillers, the only possible treatment is surgical filler removal, followed by procedures to minimize fibrosis, the deficit of volume, and any resulting deformity [7, 8].

This study focused on PMMA fillers, which are hydrophobic permanent biphasic fillers for soft tissue augmentation. PMMA microspheres are suspended in either collagen, like in Artecoll/ArteFill® (Suneva Medical, San Diego, CA), or methylcellulose, like in MetaCrill (Nutricell Laboratorios, Rio de Janeiro, Brazil) [3]. In the USA, Bellafill® (Suneva Medical, San Diego, CA) is the only FDA-approved filler for correcting atrophic acne scars, wrinkles and skin folds [9, 10].

Early adverse events after PMMA injections include erythema, swelling, and itching. However, PMMA fillers have been reported to cause, in particular, delayed and late adverse effects. Limongi et al. [11] identified 11 cases of complications of PMMA injections to the midface, which started from two to 24 months after the injection. Oedema, erythema, and contour irregularity were seen in 100% of their patients, followed by nodules (64%), yellowish xanthomatous pigmentations (36%), and eyelid malposition (18%). Histopathology demonstrated an ongoing inflammation with giant cells. Corticosteroid injection was of minimal effect. Surgical removal was performed in 82% of cases and resulted in an improvement of oedema, erythema, and nodules.

PMMA fillers have the potential to elicit a cellular immune response in humans, which consists of the development of late and ongoing inflammation and the formation of granuloma or nodules. The mechanisms at the basis of this type of immune response have yet to be well understood. One factor contributing to adverse events is the type and localization of injection. In their study on animals, Medeiros et al. [12] highlighted how submucosal injections were prone to nodularity in contrast to subcutaneous injections.

Other less frequent side effects are infections, foreign body, and biofilm formation [13].

Considering these data and the incidence of adverse events after PMMA filler, new methods could be of interest to remove them from the tissue. The aims of this work were (1) to analyse the morphology of the material in the nodules, (2) to demonstrate that treatment by acoustic wave therapy (AWT) can help remove nodules induced by PMMA injection, and (3) to present an easy procedure to solve deformities.

## Materials and Methods

### Patients

In this retrospective study, the data about 9 female patients treated for adverse effects correlated to PMMA injections in the face and body were reported. The patients were treated between April 2021 and May 2024 at the De Clinic in Milan, Italy. All patients provided informed consent for the treatments. The subsequent study was performed according to the Declaration of Helsinki’s standards for biomedical research on human subjects, and all patients provided informed consent for the analysis and publication of their data.

The adverse late events observed in these patients were pain, inflammatory reactions, erythema, nodules and granulomas, deformities, and fibrosis with functional limitations. In 6 patients, the PMMA filler was located in the face, and in 3 patients, in the body (buttocks and hands).

All patients reported previous local treatments, including steroids and 5-fluorouracil injections, and direct aspiration of PMMA. However, the response to all these therapies was very poor.

All patients underwent pre-operative assessment, including autoimmune evaluation. The latter was excluded. No patients were in therapy with non-steroidal anti-inflammatory (NSAIDs) and/or steroid drugs.

### Acoustic Wave Therapy

All patients underwent 6 sessions of AWT in the area of inflammation, 2 sessions a week for 3 weeks before surgery. For the treatment, acoustic waves were administered by the DUOLITH® SD1 ULTRA system (Storz Medical AG, Tägerwilen, Switzerland).

The system produces a vibrating massage through compressed air on targeted tissue, determining the generation and propagation of acoustic waves in this tissue. The system consists of a control unit, a pneumatically driven handpiece with multiple types of transmitters, and a pressurized air source. The pulses are generated in a ballistic way by accelerating a projectile with pressurized air, which strikes a stationary surface, which is the vibration transmitter. The generated vibrations are the radial acoustic waves that propagate directly into the tissue to treat [14].

The treatment was performed with an energy range of 2–4 bar.

### Surgeries for Polymethylmethacrylate Removal and Deficit Compensation

After the 6 sessions of AWT, the PMMA removal surgeries were performed in tumescent anaesthesia after subcutaneous infiltration of a solution containing lidocaine, adrenaline, bicarbonate, and cold saline solution.

The PMMA was removed by direct aspiration by an 18 gauge needle connected with a 10cc Luer Lock syringe.

**Fig. 1:**
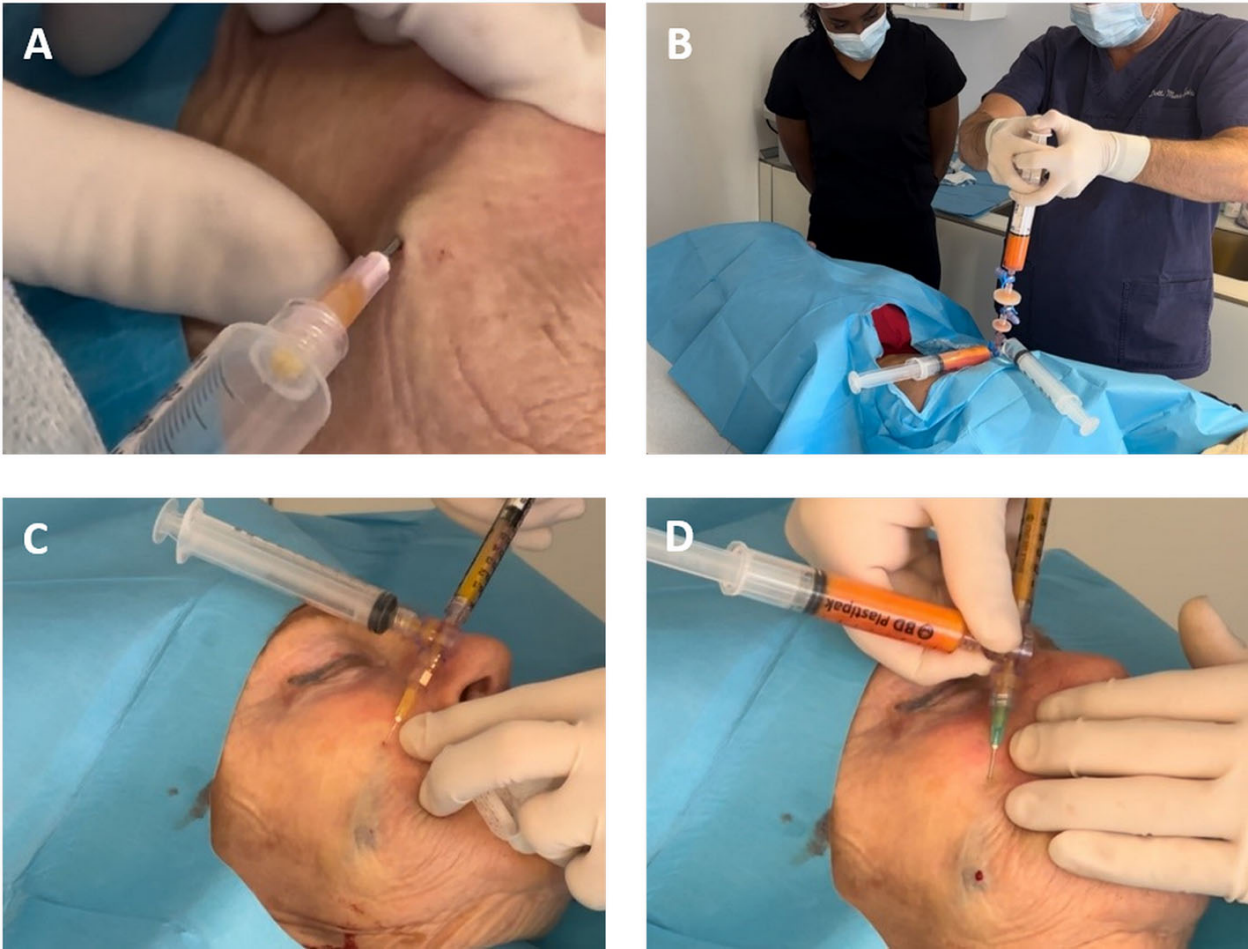
Surgical steps. PMMA mass was aspirated (**A**). Subsequently, subcutaneous fat and deep dermal filler were harvested and processed in a closed system (**B**). The enriched nanograft (**C**) and the dermagraft (**D**) were injected intradermally and in the subcutaneous layer, respectively

After aspiration, in order to fill the lack of volume related to the permanent filler removal, a graft of the deep dermis was performed, according to the dermagraft procedure (Go Easy SRL, Milan, Italy). Some deep dermal tissue was harvested from the patient's abdomen. This tissue, the fibrotic tissue mixed with dermal fat, and the superficial subcutaneous fat were processed by the closed GoEasy system (Go Easy SRL, Milan, Italy) to obtain the so-called enriched nanograft, which is an emulsion rich in adipose-derived stem cells (Fig. 1). This product was injected intradermally into the depression left from PMMA in order to promote an intense bio-revitalization of the tissue and solve the lack of volume.

### Follow-up

Patients were monitored 1 week, 6 months, and 12 months from the treatment. One patient underwent an ultrasonographic control at 38 months of follow-up. The device used was integrated in the DUOLITH® SD1 ULTRA system (Storz Medical AG, Tägerwilen, Switzerland), and the probe was of 15MHz.

### Removed Material Analysis

The leftover aspirated material was analysed by histology and scanning electron microscopy (SEM).

After being fixed with paraformaldehyde at 4% (Boster Biological Technology Co., Ltd.) for 20 minutes, samples derived from the different procedures were washed with PBS 1X, dried, and embedded with optimal cutting temperature compound (OCT). Samples were cryo-sectioned in 14-µm thick transversal slices. Then, slides were dried underflow for 30 minutes and stored at -20°C for the subsequent histological analysis. To evaluate the morphology of the samples, slides were rehydrated with distilled water and then dehydrated with increasing alcohol concentration (80–95–100% for 5 min each, and xylene twice for 5 min). Finally, a drop of mounting medium (Entellan) was added, and the slides were covered with the cover slice. All slides were examined under an Olympus BX-51 microscope (Olympus, Tokyo, Japan) equipped with a digital camera and a 40X objective (DKY-F58 CCD JVC, Yokohama, Japan).

The samples were studied by SEM for a broad morphological understanding. The sample was fixed with glutaraldehyde 2% diluted in 0.1 M phosphate buffer (pH 7.4) for 2h at 4 °C and post-fixed in 1% osmium tetroxide (OsO4) diluted in 0.2 M potassium hexacyanoferrate for 1h at 4 °C. The samples were dehydrated in a graded concentration of ethanol, followed by a critical point dryer (CPD030, Balzers, Vaduz, Liechtenstein), mounted to stubs with colloidal silver, sputtered with gold by a MED 010 coater (Balzers), and examined with FEI XL30 scanning electron microscope (FEI Company, Eindhoven, The Netherlands).

## Results

### Procedure Results

**Fig. 2:**
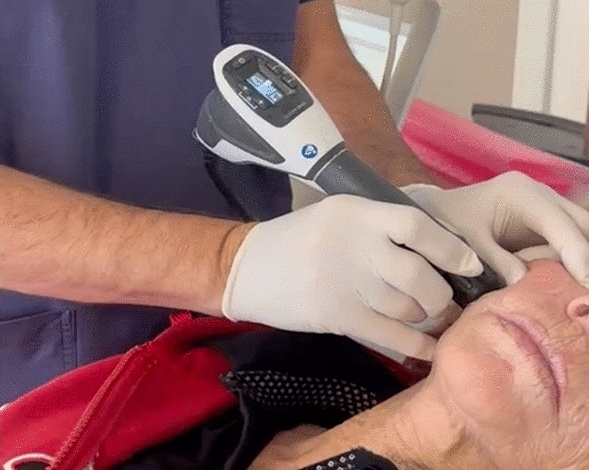
AWT before surgical removal of a PMMA permanent filler at the right cheek level

For all patients, AWT was performed without complications (Fig. 2).

The subsequent main surgical steps are schematized in Figure 1. The procedure had to be repeated twice in 1 patient and thrice in another to obtain optimum results. Minor complications, such as bruising, were observed in 5 cases after the surgeries. No patient reported infection, nerve damage, or necrosis. The average follow-up was 18 months, and no patient had relapses or subsequent complications.

**Fig. 3:**
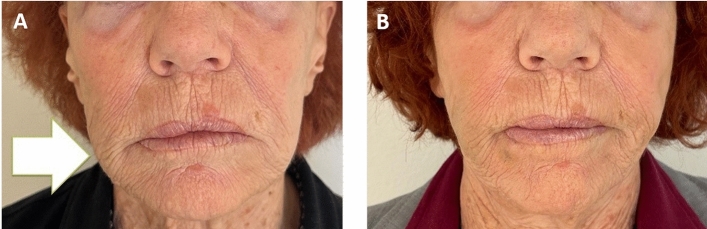
Pre (**A**) and post-treatment (**B**) views. Pre-treatment, the patient presented recurrent inflammation, pain, redness, and deformity at the right cheek level (white arrow). 10 months after the treatment, the woman's natural appearance was restored

All the patients were delighted with the aesthetic outcome (Fig. 3). In all patients, an important improvement of the deformities, with good correction of the volume of the face, was observed. A substantial resolution of the initial clinical symptoms (redness, pain, and nodules) was documented.

**Fig. 4:**
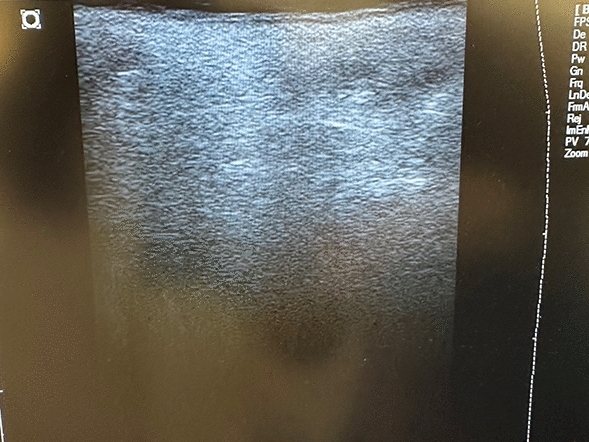
Ultrasonography of the gluteal area of a woman with a follow-up of 38 months from the PMMA removal. The tissues appear homogeneous. No PMMA residuals or fibrosis are present

The analysis of the ultrasonography performed on a single patient highlighted the complete removal of the PMMA and the resolution of the initial fibrotic condition (Fig. 4).

### Material Analysis

**Fig. 5:**
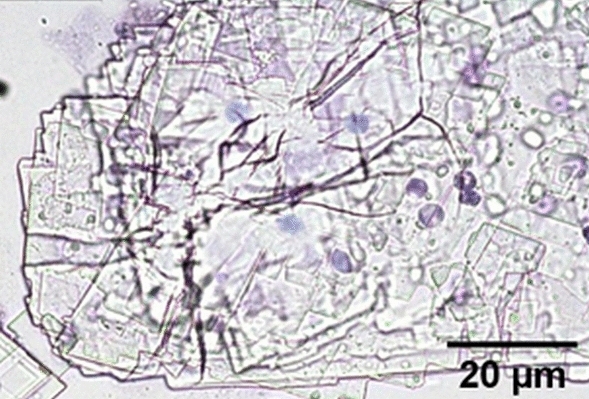
Histological image of the aspirated material. Magnification ×40

The histological analysis showed the presence of a plastic foreign body in the samples (Fig. 5).

**Fig. 6:**
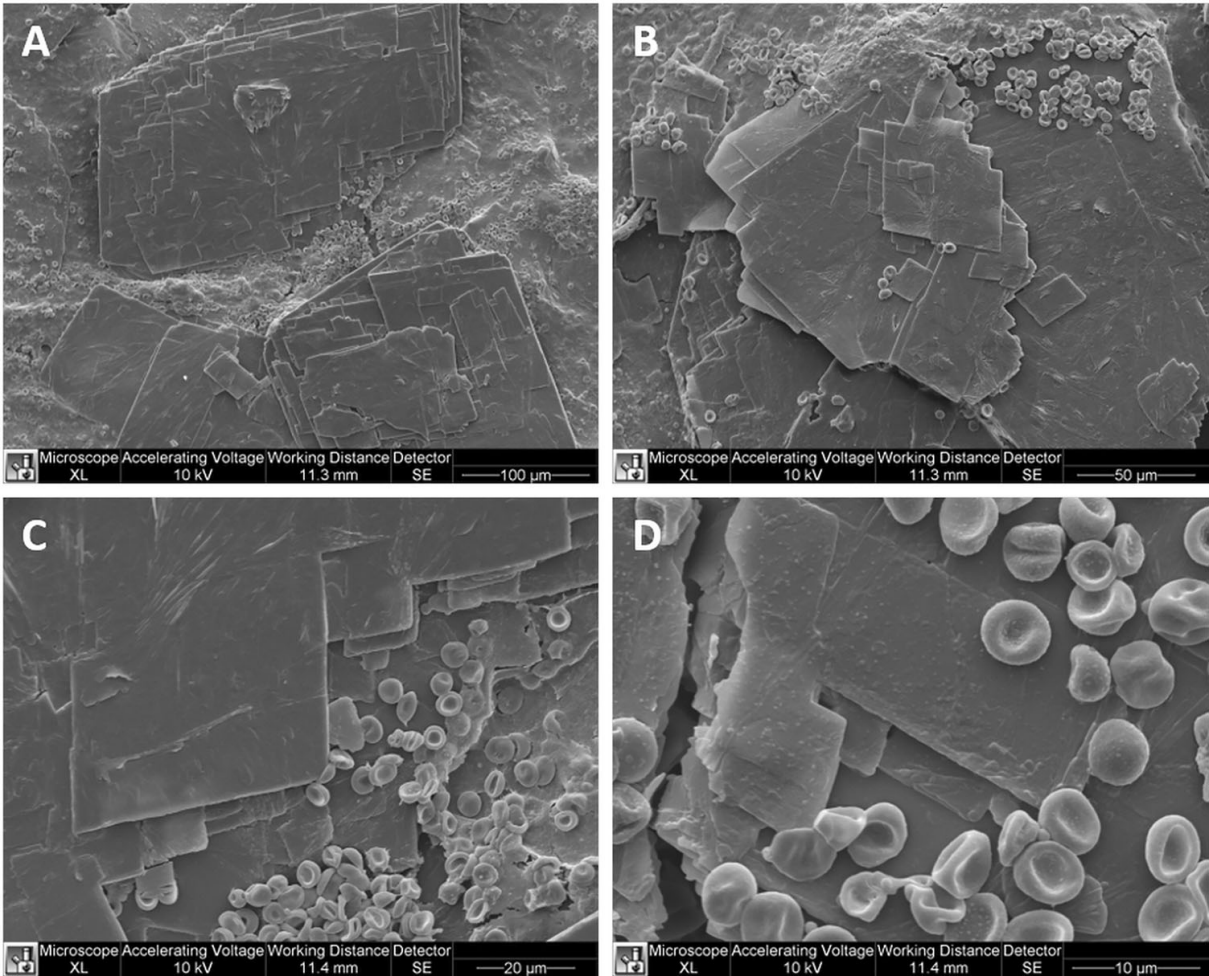
SEM analysis of the material (**A**, **B, C, D**)

The SEM analysis highlighted the micro-fragmentation of the material (Fig. 6). PMMA appears in the form of laminar or prismatic formations with a paracrystalline structure, with dimensions ranging from a few tens to a few hundred microns. This material had regular edges and corners cut at 90° with smooth surfaces. The arrangement appeared in overlapping sheets. PMMA was embedded in a mass of finely granular tissue in which blood elements were evident.

## Discussion

The occurrence of side effects correlated to PMMA filler is low [15]. Nonetheless, cases related to PMMA filler and, also, to other permanent fillers have been described in the literature [15–19]. Park et al. [16] proposed an algorithm for the management of complications with treatments ranging from topical steroid application in case of allergic reaction to surgical excision in case of nodule or granuloma formation.

Generally, to solve granulomas, first of all, injection of 5-fluorouracil and triamcinolone is suggested. Second, in case of unsuccess of this type of injection, steroids and hyaluronidase injections are recommended [17]. In case of no improvement, a treatment option is removing the material by intralesional laser treatment [7, 20–22]. The first to use the intralesional laser to manage filler complications was Cassuto et al. [20], by the 808 nm diode laser at 6 to 8W with a pulse duration of 500 ms to 1000 ms. He described the treatment of 219 patients, with complete disappearance of nodules and lumps in 62% of subjects and partial improvement in 30%.

During laser contact with the polymer surface, the temperature increases rapidly till the melting point of the polymer [7]. For this reason, the complications of this procedure can be burning, post-inflammatory hyperpigmentation, sterile abscess formation, and impairment of facial nerves. In any case, the thermal injury creates scar tissue in the area of laser application.

When all the other treatment options fail, the last possibility is to resort to traditional plastic surgery, which is, however, a challenging option to remove PMMA. Fibrosis and subcutaneous tissue scarring involving the vascular and nervous plexus can lead to catastrophic complications.

Durkin et al. [17] and Park et al. [16] described successful surgical cases. In particular, Durkin et al. [17] reported 3 cases in which the blepharoplasty approach was used to remove PMMA filler from the submalar area. They underlined how the procedure is time intensive, technical demanding, and lasted, on average, between 3 and 6 h. Parks et al. [16] recommended the resort to surgery as the last step.

Acoustic shock waves (ASWs) were first applied as extracorporeal shock wave therapy (ESWT) to PMMA in some experimental studies of total hip arthroplasty revision in dogs and pigs. Arthroplasty revision surgery has a significant morbidity, which can be partially attributed to PMMA cement. The ASWs were described as facilitating the extraction of PMMA [22–25].

Hach et al. [26] demonstrated an evident decrease in the force necessary for extracting bone cement after applying shock waves. They found no macroscopically evident damage to the cortical bone and the surrounding tissues.

Shock waves were used in vivo for the disintegration of renal and ureteral calculi in 1980. The therapy was named extracorporeal shock wave lithotripsy. Shortly after that, in 1985, its use was extended to the rupture of gallbladder stones. In 1988, the use of acoustic shock waves for the treatment of pseudarthrosis of long bone fractures was successfully tested in Germany. In the 1990s, they were applied to treating plantar fasciitis, lateral epicondylitis (tennis elbow), and calcific tendonitis of the rotator cuff. Application fields multiply over the years.

Although the breakdown of urinary and gallstones is purely mechanical, low-energy shock waves have shown a mechanotransduction effect on the biochemistry of the cell. This aspect permits us to understand the healing powers of acoustic shock waves in various tissues [27, 28]. In fact, when applied in non-urological indications, the mechanisms of action of shock waves are not linked to the direct mechanical effect but to the different types of biological reactions induced by acoustic stimulations through mechanotransduction. Mechanotransduction is a particular biological response of many cell types, which involves the conversion of biomechanical forces from the extracellular environment into biochemical responses after the perception of these biomechanical forces and the processing of the resulting signalling. The biochemical reactions consist of the modulation of some fundamental cellular functions such as migration, proliferation, differentiation, and apoptosis.

The action mechanism is the rearrangement of proteins within the cell membrane, which induces changes in the cells’ tridimensional structure and modifies their activity according to the different biomechanical conditions.

Furthermore, some experimental models have already demonstrated that the application of acoustic shock waves to intramuscular silicone masses (created through targeted injections) reduced the formation of the typical dense fibrous capsule, deriving from the presence of a foreign body and correlated to synergistic alterations in the levels of pro and anti-fibrotic proteins (transforming growth factor beta 1, TGF-beta1, and matrix metalloproteinase 2, MMP-2, respectively). When the therapy with acoustic shock waves was applied in the form of multiple sessions, it degraded the fibrous envelope. These experiments demonstrated that shock waves could reduce capsule formation and induce remodelling/resorption of fibrotic tissue.

According to the literature, acoustic shock waves enhance not simply healing processes but proper regenerative events, for instance, reducing fibrous tissue at its origin or even remodelling it in a second phase, as in scars [27–29].

It should be emphasized that the pathogenetic mechanism of the formation of capsular fibrosis in silicone prostheses presents several similarities with the fibrotic reaction around the PMMA filler [28]. In fact, it is thought that direct immunostimulation and sub-clinical infection are the main ones responsible for the induction and maintenance of inflammatory reactions, which lead to the overabundant formation of extracellular matrix. Shock waves could also inhibit inflammatory processes, revealing antibacterial capacity [30].

Different application modalities of the therapy with shock waves induce different therapeutic results. For instance, after the insertion of silicone devices, a single application of this therapy can decelerate capsule formation in contrast to multiple therapies, which degrades fibrotic tissue [31]. Analogously, acoustic shock waves applied in multiple sessions permitted the reduction in the number of keloids’ collagen fibres, thanks to the increase in the matrix metalloproteinase MMP13 gene expression [32].

AWT was born from the application of acoustic shock waves in aesthetic and regenerative medicine. It is widely used with promising results in cellulite treatment [33–35], facial skin texture, pores, and wrinkles [36], and body shaping and fat reduction [37–39].

This is the first report on the AWT application for removing PMMA injectable fillers. The main advantage of this technique is that it does not induce any thermal damage, as happens with lasers. Moreover, it is less time-consuming and technical demanding with respect to a standard surgery. The results obtained are fascinating as, without major side effects, it permitted the resolution of the connective tissue alterations correlated with the presence of PMMA, as already documented for capsular fibrosis [31, 32]. The last ones can be compared with the well-delimited filler volume, around which fibroblasts synthesize and cause collagen deposits [40]. Consequently, AWT enters the field of non-invasive, well-tolerated, and easy-to-use therapies.

PMMA inside a tissue can take on different appearances depending on the tissue's pH, temperature, and vascularization. The histological and SEM analyses performed in this study highlighted that the precipitation of PMMA fillers occurs in paracrystalline form. These are generally overlapping laminar structures that probably interact with the host structures by attracting macrophage elements and consequent induction of fibrotic phenomena. The conformation of the material with such precise edges and sharp 90-degree angles seems unsuitable for integrating with the vascular and connective structures present. In contrast, this structure is particularly susceptible to the AWT effects.

The main limitation of this study is the small number of patients involved. For this reason, it remains to verify whether the observed phenomenon is a generalized phenomenon present in all PMMA injections or whether it is rather a local situation due to a particular reaction of the tissue. It must be added that long-term results might also be influenced by epigenetic factors.

Moreover, even if the ultrasound analysis highlighted the complete removal of PMMA and the absence of fibrosis, it referred to a single case. This result is extremely promising, but must be verified for all the patients, at the right follow-up.

The other limitation of this study is the lack of long-term follow-up because 2 months of follow-up is not a sufficient time to completely exclude the eventual recurrence of fibrosis over eventual residual filler. However, according to the literature [41], the formation of granuloma correlated with permanent fillers might occur up to 10 years after the injections and, in this case, after the removal of the eventual residual filler. Consequently, long monitoring of these patients is not only recommended but required.

Further studies with a larger cohort and with long-term follow-up are required to consolidate the technique presented in the present study.

## Conclusion

The data reported in this study highlighted how AWT acts directly on PMMA to facilitate its removal. As a second result, it reduces fibrosis around the implanted permanent filler. Finally, since the consequence of PMMA removal is a lack of volume, a graft must be performed, and AWT, by increasing tissue vascularization, also increases the survival rate of the graft.

Further studies are needed to evaluate better the use of AWT on PMMA's long-term side effects, but the findings of this study allow us to describe a new possible field of AWT application. This method could also be helpful in the removal of hard material with different compositions.
