# Correction: Extracorporeal Acoustic Shock Waves to Treat Complications of Polymethylmethacrylate

**DOI:** 10.1007/s00266-025-04711-4

**Published:** 2025-02-04

**Authors:** Mario Goisis, Sara Zecchetto, Sheila Veronese, Lindsey Alejandra Quintero Sierra, Riccardo Ossanna, Paolo Bernardi, Maria Maddalena Nicoletti, Sima Khabouri, Andrea Sbarbati

**Affiliations:** 1 De Clinic, Viale Regina Giovanna 39, 20129 Milan, Italy; 2 Aesthetic Medicine Clinic - Dr. Sara Zecchetto, Piazza del Popolo 16, 37132 Verona, Italy; 3 Section of Anatomy and Histology, Department of Neuroscience, Biomedicine, and Movement, University of Verona, P.le L.A. Scuro 10, 37134 Verona, Italy; 4 Department of Precision Medicine, University of Campania “Luigi Vanvitelli”, Piazza Luigi Miraglia 2, 80138 Naples, Italy

**Correction to: Aesth Plast Surg** 10.1007/s00266-024-04586-x

The article “Extracorporeal Acoustic Shock Waves to Treat Complications of Polymethylmethacrylate”, written by Mario Goisis et al, was originally published under exclusive license to Springer Science+Business Media, LLC, part of Springer Nature and International Society of Aesthetic Plastic Surgery. As a result of the subsequent decision to publish the article under the open access model, the article’s copyright notice was changed on 16 January 2025 to © The Author(s) 2024 and the article is now distributed under a Creative Commons Attribution CC BY. This licence permits use, duplication, adaptation, distribution and reproduction in any medium or format, as long as appropriate credit is given to the original author(s) and the source, a link is provided to the Creative Commons licence, and any changes are indicated. Please read the full licence for further details at-http://creativecommons.org/licences/by/4.0.

